# You are what you eat, and more

**DOI:** 10.1042/EBC20254001

**Published:** 2025-12-23

**Authors:** Caroline Lei Wee

**Affiliations:** 1 Institute of Molecular and Cell Biology (IMCB), Agency for Science, Technology and Research, A*STAR, 61 Biopolis Drive, 138673, Singapore

**Keywords:** Gut Microbiome, Gut Microbiota, Microbial Metabolites, Dysbiosis

We have all heard of the age-old saying, ‘You are what you eat’. With the rapid rise of microbiome research, evidenced by the exponential increase in publications on this topic over the past decade (Figure 1), it is now clear that health and disease are shaped not only by our diet but also by the trillions of microbes residing within our guts. Hence, the phrase might now be better expressed as: ‘You are what you – *and your gut microbes* – eat’.

**Figure 1:**
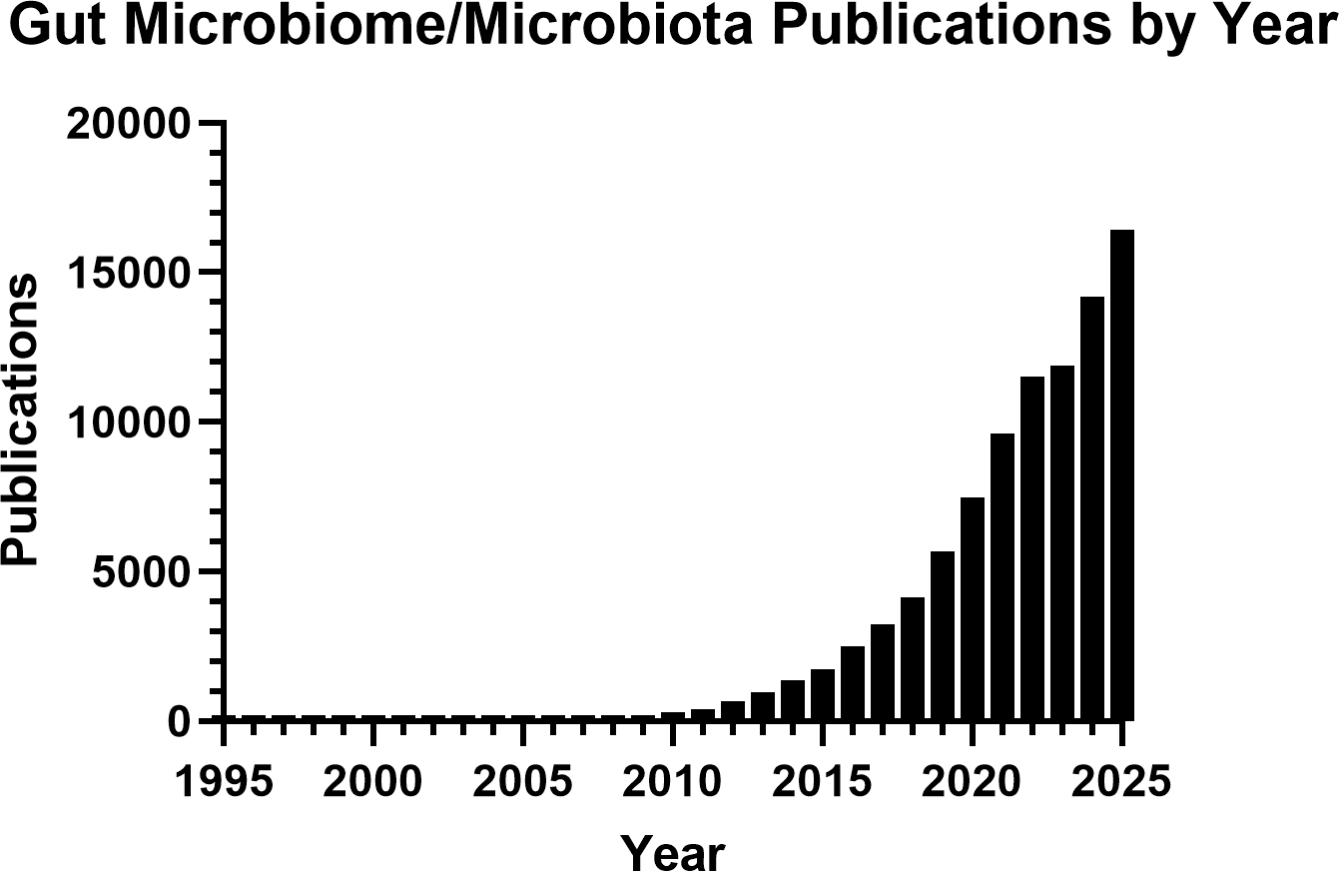
Number of PubMed journal articles containing ‘gut microbiome’ or ‘gut microbiota’ in the title or abstract from 1995 to 2025.

Indeed, the microbiome community in the human gastrointestinal (GI) tract has the incredible capacity to ferment dietary nutrients, producing metabolites that have broad physiological effects – either by acting within the gut, or via absorption into the circulation [1]. Hormone-releasing cells and neuronal connections within the gut may be activated by microbes and their metabolites, signaling to other organs such as the brain [2]. Diet or lifestyle changes, infections, medications, and other environmental or physiological factors are capable of shifting gut microbiome composition, in turn affecting its metabolic capacity and downstream signaling pathways [3]. Additionally, such perturbations to the gut microbial community may trigger immune responses or even damage gut barrier integrity, leading to the entry of microbial components or pathogens into the circulation [4]. It is therefore not surprising that these perturbations may influence diseases of, and beyond the gut, and also modulate responses to therapeutics [4]. Clearly, it is important to examine the causal mechanisms linking microbial homeostatic functions or imbalance (‘dysbiosis’) to disease and therapeutic outcomes.

Articles in this special issue of *Essays in Biochemistry* explore the homeostatic functions of the microbiome (Beretta and Schwab, in preparation), specifically how fermentation metabolites such as short-chain fatty acids, alcohols, and amino acid metabolites are produced along the GI tract, as well as how fermentation capacity can be restored after dysbiosis. One class of metabolites derived from the amino acid tryptophan, known as indole derivatives, is associated with immune and metabolic health and disorders. Dormans et al. (in preparation) review how these metabolites may influence cardiovascular disease and cancer risk by modulating inflammatory pathways affecting atherosclerosis and tumor growth. Similarly, these metabolites may improve cancer immunotherapy efficacy or reduce its cardiotoxic side effects; hence, indole-based interventions could potentially be harnessed for better therapeutic outcomes. Furthermore, Park, Nagpal et al. (in preparation) propose that gut microbial dysbiosis may be a hallmark and driver of neurodegenerative disorders such as Alzheimer’s disease via diverse gut–brain signaling mechanisms, again suggesting that restoring gut microbial homeostasis could be key to mitigating cognitive decline over aging.

Together these review articles cover the present state of the field, and highlight fundamental questions that remain unresolved. Establishing specific mechanisms underlying gut microbiome–host interactions remains a challenge, as they are highly complex and incompletely understood. Given the diversity of the gut microbiome and metabolome even among humans, these articles also propose personalized prevention and therapeutic strategies to maximize health outcomes. Overall, due to its extensive impact on human biology, the gut microbiome is a highly promising target for the detection, prevention, and treatment of disease.

## Open Access

This article has been published open access under our Subscribe to Open programme, made possible through the support of our subscribing institutions, learn more here: https://portlandpress.com/pages/open_access_options_and_prices#conditional
